# Analysis of erroneous data entries in paper based and electronic data collection

**DOI:** 10.1186/s13104-019-4574-8

**Published:** 2019-08-22

**Authors:** Benedikt Ley, Komal Raj Rijal, Jutta Marfurt, Naba Raj Adhikari, Megha Raj Banjara, Upendra Thapa Shrestha, Kamala Thriemer, Ric N. Price, Prakash Ghimire

**Affiliations:** 1 Global and Tropical Health Division, Menzies School of Health Research and Charles Darwin University, Darwin, Australia; 2 Central Department of Microbiology, Tribhuvan University, Kirtipur, Kathmandu Nepal; 3 Mahidol-Oxford Tropical Medicine Research Unit (MORU), Faculty of Tropical Medicine, Mahidol University, Bangkok, Thailand; 4 Centre for Tropical Medicine and Global Health, Nuffield Department of Clinical Medicine, University of Oxford, Oxford, UK

**Keywords:** Electronic data entry, Paper based data entry, AKVO, Epidata

## Abstract

**Objective:**

Electronic data collection (EDC) has become a suitable alternative to paper based data collection (PBDC) in biomedical research even in resource poor settings. During a survey in Nepal, data were collected using both systems and data entry errors compared between both methods. Collected data were checked for completeness, values outside of realistic ranges, internal logic and date variables for reasonable time frames. Variables were grouped into 5 categories and the number of discordant entries were compared between both systems, overall and per variable category.

**Results:**

Data from 52 variables collected from 358 participants were available. Discrepancies between both data sets were found in 12.6% of all entries (2352/18,616). Differences between data points were identified in 18.0% (643/3580) of continuous variables, 15.8% of time variables (113/716), 13.0% of date variables (140/1074), 12.0% of text variables (86/716), and 10.9% of categorical variables (1370/12,530). Overall 64% (1499/2352) of all discrepancies were due to data omissions, 76.6% (1148/1499) of missing entries were among categorical data. Omissions in PBDC (n = 1002) were twice as frequent as in EDC (n = 497, p < 0.001). Data omissions, specifically among categorical variables were identified as the greatest source of error. If designed accordingly, EDC can address this short fall effectively.

**Electronic supplementary material:**

The online version of this article (10.1186/s13104-019-4574-8) contains supplementary material, which is available to authorized users.

## Introduction

Paper-based case report forms (CRF) are the most widely used form of data collection in field-based research studies. In this approach data collected are recorded on paper and subsequently digitalized. While straight forward to implement, paper-based data systems risks introducing errors both at the time of data collection and during digitalization.

Electronic data collection (EDC) has become a well-accepted alternative to paper-based data collection (PBDC), reducing the risk of errors and allowing real time checks for completeness and logical consistency [1–3].

Both PBDC and EDC are prone to inaccuracy through erroneous data entry and data omissions [4]. Data collected during a hospital-based survey in Nepal were recorded on paper and in electronic form. This article analyses the patterns in data entry errors among both systems.

## Main text

### Procedures

The study was conducted at a hospital in Danghadi, in the East of the country. Patients were screened and eligible patients invited to participate. Following written informed consent, each participant was assigned a study identification number (CODE). A questionnaire was completed and a brief physical examination made, the results of which were recorded in the presence of the patient. Venous blood was collected and shipped to a collaborating laboratory for subsequent analysis. Data were generated and recorded at two time-points, in the presence of the patient and in the laboratory. For EDC, two distinct databases were created one for data collected with the patient, one for the corresponding laboratory data; for the PBDC, all data were recorded on the same CRF and later entered into a single database. The same staff performed PBDC and EDC, all had at minimum a high-school degree, none had previous experience in electronic data collection.

The EDC system was designed using open-source software from AKVO (NL; https://akvo.org/, last accessed on 11.07.2019). Data were entered on tablets (Galaxy Tab a 7.0 SM T285, Samsung, Seoul, South Korea). Each study participant was assigned with two identifiers: a database specific unique identifier provided automatically through the system, and another assigned by the data collector (CODE). The system did not check for the uniqueness of the assigned CODE. During data entry, the custom-made data entry mask performed completeness and range checks, erroneous results were indicated to the data entry clerk at time point of entry, however, data entry could proceed even in case of unresolved errors. The system could not detect logic errors nor calendar related errors, such as enrolment before date of birth. All entered data were uploaded immediately upon data entry via Wi-Fi into a cloud based database and could be monitored by a third person in real time.

For the PBDC data entry, CRFs were completed and digitalized within 7 days using EpiData version 3.1 (EpiData Association, Odense, Denmark) [5]. This version is no longer supported, however offers the possibility for logic, range, and date checks. The data entry system was designed so that CODE could not be assigned more than once. In all other cases, the system highlighted detected errors during data entry by prompting the data entry clerk through a pop-up window and an acoustic signal. Like the EDC, data entry could proceed despite the erroneous entry. While technically possible, the system was not designed to prompt the data entry clerk in case a variable was not entered.

### Data analysis

Once data entry was completed, digitalized data were exported to Excel (Microsoft, Redmond, USA) and the hospital and lab database from EDC were merged using the patients’ identifier (CODE). The following errors were checked: completeness of variable entry, values outside of realistic ranges, internal logic violations in a subset of variables, and dates outside a reasonable time frames. In the absence of a reference method, results were compared from both databases using CODE as the matching variable. The database was rebuilt coding concordant variable entries as “0” and discordant entries as “1”. Each variable was categorized as text, continuous, categorical, date, or time variables.

Statistical analysis was done using Stata version 14 (Stata Corp, USA). Differences in proportions were assessed using Chi square test and the McNemar’s test for correlated proportions as appropriate.

The number of discordant entries were compared between both systems, overall and per variable category, and whether the proportion of discordant results differed between lab and field data entry. Among discordant results, missing entries and out of range results were quantified and compared between both systems. Missing data were assessed for missing data mechanisms, the only patient specific variable included was sex [6]. Among non-missing discordant continuous variables, the absolute difference was calculated between both entries and expressed as a fraction of the higher entry in percent (%). As a proxy for logical errors among discordant results, we determined whether date of birth was after date of enrolment and calculated the body mass index (BMI) from body weight and height for all participants [7]. A BMI above 40 or below 12 was defined as unrealistic irrespective of the participants age.

### Results

Data were collected from 362 patients. The EDC database contained a duplication of the unique identifier (CODE) in two cases, accordingly four participants were excluded from both databases, resulting in 358 (98.9%) participants with paired data collected by both systems. Each data set contained 56 variables (Additional file 1: Table S1). A total of 4 (7.1%) variables were excluded from this analysis: the variable “CODE” as this was the linking variable between datasets, “Study Name” (STUDY) since this was autocompleted among both systems, “Patient Initials” (INI) and “Place of Birth” (POB) as these were entered as free text and had to be translated from Devanagari to Roman letters. From the 52 (92.9%) included variables, a total of 3 (5.8%) variables contained dates, 2 (3.8%) variables recorded a specific time (in 24-h format), 10 (19.2%) variables contained continuous data, 35 (67.3%) variables contained categorical data and 2 (3.8%) variables contained text where it was assumed that data collectors would know the correct spelling of all possible answers in Roman letters (“Diagnosis-DIAG” and “Main Place of Residence-MPR”, Additional file 1: Table S1).

**Fig. 1:**
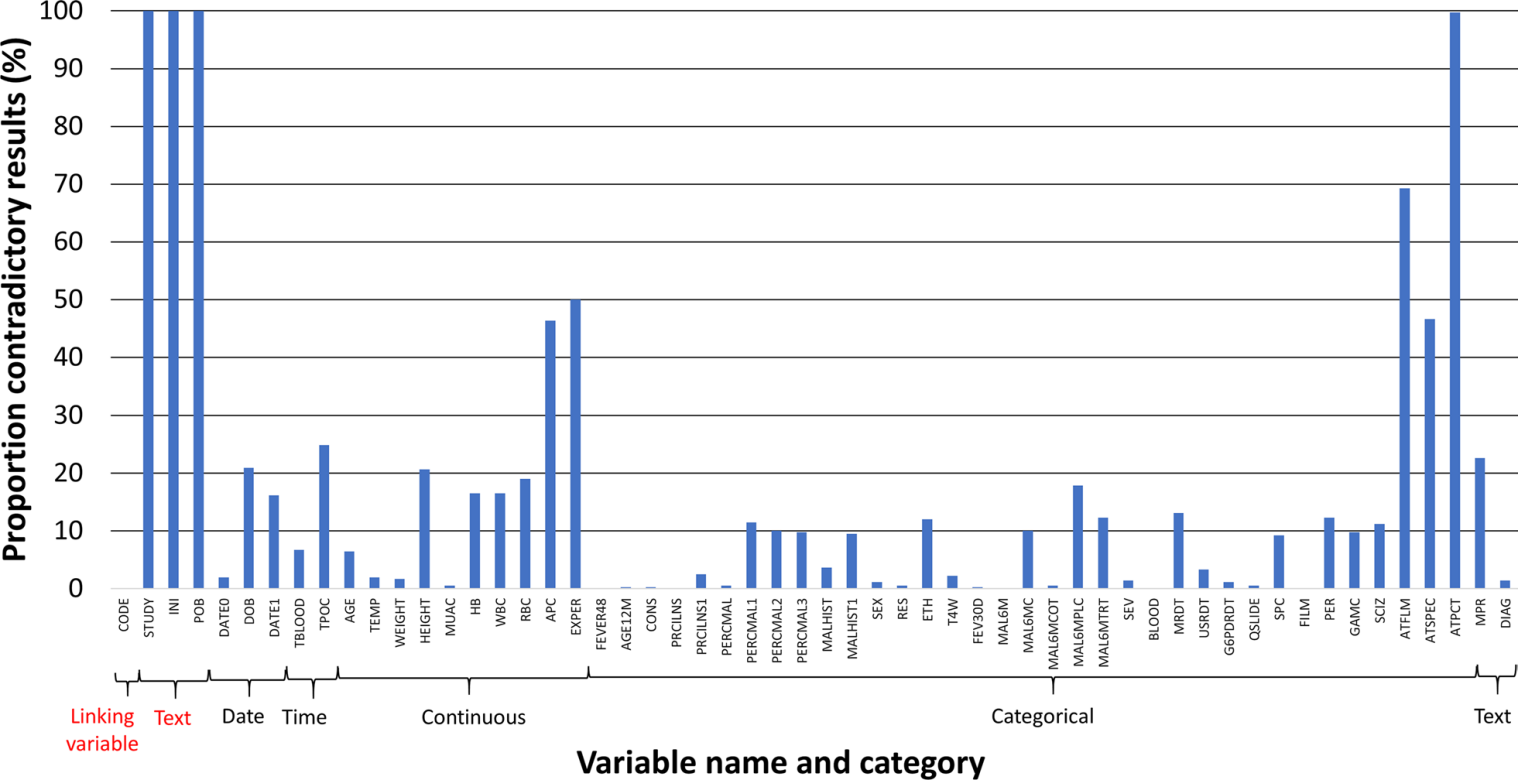
Proportion of discrepant results per variable and variable category. Red highlighted variables are excluded from analysis

Discrepancies between both data sets were found in 12.6% (2352/18,616) of all entries, with differences between databases detected in 18.0% (643/3580) of continuous variables, 15.8% (113/716) of time variables, 13.0% (140/1074) of date variables, 12.0% (86/716) text variables, and 10.9% (1370/12,530) of categorical variables (Table 1 and Fig. 1).

**Table 1:** Overview of discrepant results among all data entered, and among discrepant results: number of blanks, out of range entries and logical errors

Variable type	No. of variables	All	Among contradictory entries								
		Contradictory entries/total (%)	Blanks in Epidata/total (%)	Blanks in AKVO/total (%)	p blanks	Epidata: out of range entries/total (%)	AKVO: out of range entries/total (%)	p out of range entries	Epidata: logical errors/total (%)	AKVO: logical errors/total (%)	p logical errors
Date	3	13.0% (140/1074)	0.0% (0/140)	52.9% (74/140)	*< 0.001*	0.0% (0/140)	0.7% (1/140)	0.317	0.0% (0/140)^a^	0.7% (1/140)^a^	0.317
Time	2	15.8% (113/716)	0.0% (0/113)	0.0% (0/113)	1.000	0.0% (0/113)	0.0% (0/113)	1.000	NA	NA	–
Categorical	35	10.9% (1370/12,530)	60.1% (823/1370)	23.7% (325/1370)	*< 0.001*	NA	NA	–	NA	NA	–
Continuous	10	18.0% (643/3580)	26.9% (178/643)	14.1% (91/643)	*< 0.001*	0.2% (1/643)	0.0% (0/643)	0.317	0.0% (0/643)^b^	0.2% (1/643)^b^	0.317
Text	2	12.0% (86/716)	1.2% (1/86)	8.1% (7/86)	*0.034*	NA	NA	–	NA	NA	–
Total	52	12.6% (2352/18,616)	42.6% (1002/2352)	21.1% (497/2352)	*< 0.001*	0.1% (1/896)	0.1% (1/896)	1.000	0.0% (0/783)	0.1% (1/783)	0.318

^a^Counting all cases with date of birth after date of enrolment

^b^Counting all cases with a BMI < 12 or BMI > 40 among discrepant results

A total of 64% (1499/2352) of all discordant entries were due to data being entered in one system, but not the other. The largest proportion of omissions was among categorical variables (76.6%, 1148/1499), followed by continuous variables (17.9%, 269/1499), dates (4.9%, 74/1499, all in the EDC) and text variables (0.5%, 8/1499). Overall 66.8% (1002/1499) of data were omitted from PBDC/Epidata method compared to 33.2% (497/1499) of data entered in the EDC database (p < 0.001). Significantly higher proportions of omissions were found in the PBDC database among categorical and continuous variables, date and text variables had significantly higher proportions of missing entries in the EDC database (all p < 0.05) (Table 1). When considering data omissions that were found in both systems, the proportion of missing data was slightly higher among females (n = 1949/5695, 25.5%) compared to males (n = 2521/8451, 23.0%), p < 0.001.

While designed to tolerate data omissions, this was not desirable. Depending on variable format, entries “0”, “9” and “99” should have been entered in case data was not available, a question was not relevant or a test result was negative. In 42% (624/1499) of all discordant blanks found in the PBDC database only, a respective entry was found in the EDC database, while the opposite was the case in 3% (44/1499) of records (p < 0.01). Among discordant entries, one date entry in the EDC database and one continuous variable in the PBDC database were found to be out of range, one logical error among discordant date variables and one among discordant continuous variables was found within the EDC database (Table 1).

Among the 10 continuous variables, the median relative difference between entries ranged from 1.0% (interquartile range (IQR): 0.39–1.00, range 0.20–1.94) for measured body temperature (TEMP) to 55.1% (IQR: 34.09–76.46, range 2.44–95.33) for malaria parasite count per white blood cells (APC).

**Fig. 2:**
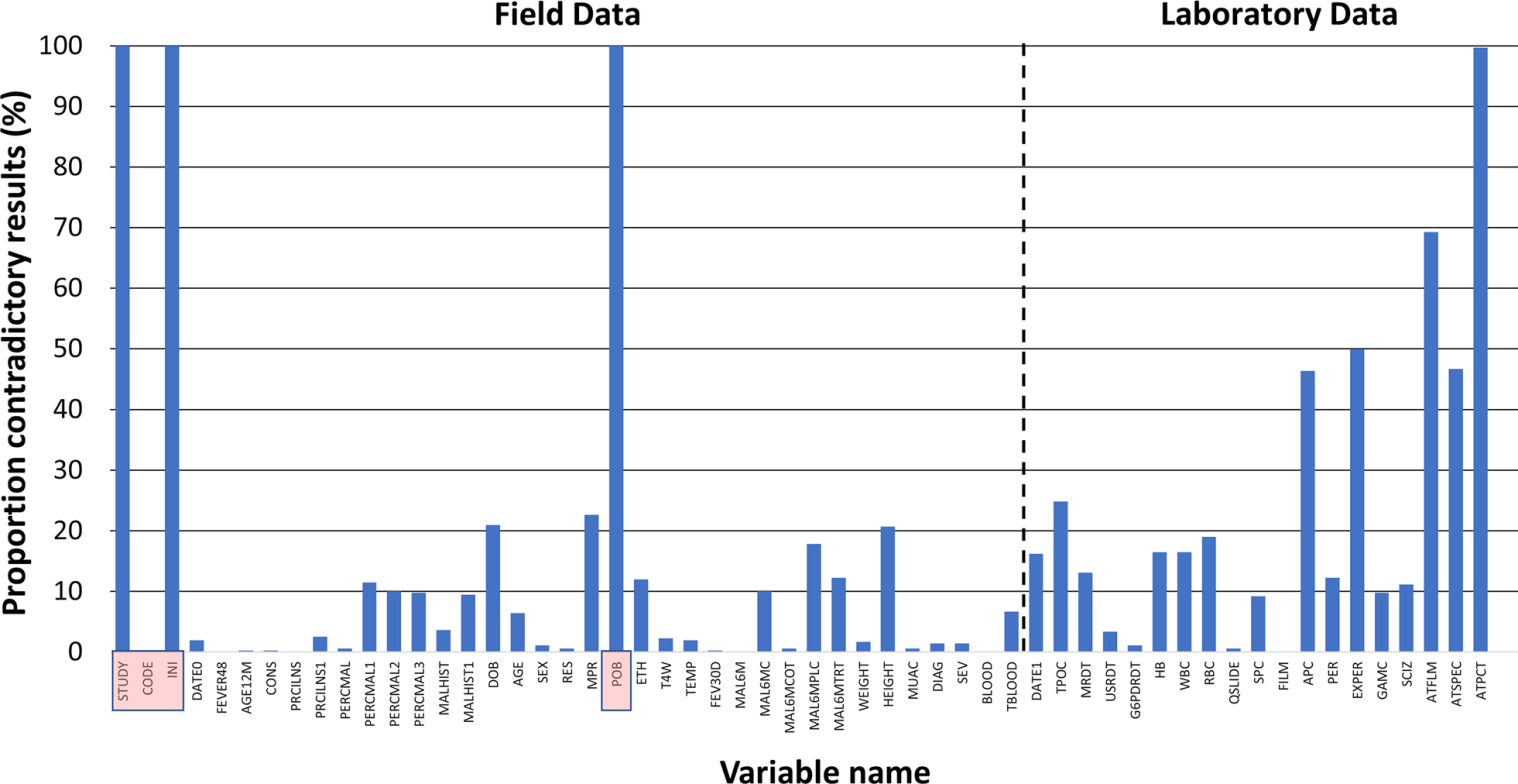
Proportion of discrepant results per variable sorted in sequence of data collection. Variables left of the dotted line are collected with the patient, variables right of the dotted line are collected in the laboratory. Red highlighted variables are excluded from analysis

A total of 33 variables were collected with the patient present, with discordant entries present in 5.8% (685/11,814), significantly fewer (p < 0.001) than among the 19 variables collected in the laboratory where 24.5% (1667/6802) of all entries differed between both systems (Fig. 2). The observed difference was applicable to all relevant categories (all p < 0.05), with 3.2% (374/11,814) blanks generated for the patient data and 16.6% (1130/6802) for the laboratory data; p < 0.001.

### Discussion and conclusion

When comparing both data entry methods, discrepant results were present in more than 12% of all entries, with almost two-thirds (64%) of these errors due to data omissions. The distribution of data omissions varied significantly across different categories. Data not entered in either system suggest that these had not been collected in the first place, and the proportion of these was slightly but significantly higher among female participants. Considering the differences in data omissions per variable category and the overall difference in data not being collected by gender suggest that data collection suffered from a “missing not at random” (MNAR) pattern [6]. Both systems provided the possibility to enter a negative response reflecting that data had not been collected rather than leaving variables blank, however this was frequently not applied and at significantly greater proportion in the PBDC system. Data omissions were found most frequently among categorical data, which were entered by ticking a box on the paper form and then during digitalization by entering a single digit from a drop-down menu in Epidata or by ticking a box in the AKVO system. Accordingly, categorical data are easy and fast to enter, but result in an increased risk of data omission, especially when data are entered in bulk, as was the case for the PBDC method.

Whilst continuous data showed the greatest overall rate of discrepant results, this was much more likely to be due to erroneous data entry than omission. Discrepant results varied substantially between variables. For instance, body temperature showed a median variation of 1% between both entries, whereas for the parasite count the difference rose to more than 50%. Since both systems only allow data entry within predefined ranges, the number of unrealistic results was very low. We found that range checks for continuous data were counterproductive as they masked erroneous data entry: while an erroneous date entry outside a reasonable range is detected quite easily, range checks would not permit extreme entries, masking errors. Double data entry could address this problem; however, this is neither practical nor realistic for EDC.

Epidata allowed for internal logic checks at the time of data entry, not possible in the AKVO system. The BMI was calculated and used as a proxy for logical errors, however there were very few unrealistic entries in either data systems. Dates were also checked for unlogic sequences, but this was not a major source of error. Interestingly all date omissions were found in the EDC.

An inherent problem of any EDC system is data collection for the same individual at different time points, as is the case for studies with follow up visits or studies with field and laboratory components. Where collected data cannot be uploaded directly, subsequent merging of data can be problematic. We found discordant results for variables generated in the laboratory to be significantly greater than for variables generated in the clinic. When assessing the pattern of erroneous entries in the laboratory (Additional file 1: Table S1), errors occurred in sequential blocks, which may reflect that the participant identifier (CODE) for the EDC data had been assigned erroneously in the laboratory. Alternatively, the quality of data entry may vary between the clinical and the laboratory staff, highlighting the importance for adequate training of all study personnel.

Our findings add to the growing body of evidence that suggests that EDC is an effective alternative to traditional paper-based data collection [2, 8, 9]. Although cost effectiveness was not assessed, previous studies suggest that EDC is cost effective, at least in large studies [2, 10].

In conclusion, we found the greatest source of data error was attributable to data omissions, specifically among categorical variables. Data omissions appeared to be following a MNAR pattern and this needs to be addressed in a twofold approach. A well-designed EDC system that does not permit blank entries can address omission of recorded data, extensive training of data collection staff in hindsight to the socio-cultural context is likely to improve the quality of data collection. Since direct electronic data collection is unlikely to be performed in duplicate, a system that performs real-time logic checks would be highly desirable. EDC, however, may only be suitable if data can be synchronized in real time and can be accessed from multiple locations, which requires a fairly complex preparatory phase and this may not be cost effective for small studies.

## Limitations

In the absence of a reference method we could not determine the quality of data entry per system directly. We also did not record the identity of the data entry staff per case record and accordingly could not assess differences in data entry quality per staff.

## Additional file

**Additional file 1: Table S1.** Database coded as discrepant (1) and concordant (0) data entries.

## Data Availability

All relevant data are in Additional file 1.

## Abbreviations

BMI: body mass index
CRF: case record form
EDC: electronic data collection
MNAR: missing not at random
PBDC: paper based data collection
